# Structural modifications of berberine and their binding effects towards polymorphic deoxyribonucleic acid structures: A review

**DOI:** 10.3389/fphar.2022.940282

**Published:** 2022-08-09

**Authors:** Lanlan Fu, Jiajia Mou, Yanru Deng, Xiaoliang Ren

**Affiliations:** School of Chinese Materia Medica, Tianjin University of Traditional Chinese Medicine, Tianjin, China

**Keywords:** berberine, modification, derivative, anti-tumor effect, DNA binding

## Abstract

Berberine (BBR) is a plant derived quaternary benzylisoquinoline alkaloid, which has been widely used in traditional medicines for a long term. It possesses broad pharmacological effects and is widely applied in clinical. In recent years, the anti-tumor effects of BBR have attracted more and more attention of the researchers. The canonical right-handed double-stranded helical deoxyribonucleic acid (B-DNA) and its polymorphs occur under various environmental conditions and are involved in a plethora of genetic instability-related diseases especially tumor. BBR showed differential binding effects towards various polymorphic DNA structures. But its poor lipophilicity and fast metabolism limited its clinical utility. Structural modification of BBR is an effective approach to improve its DNA binding activity and bioavailability *in vivo*. A large number of studies dedicated to improving the binding affinities of BBR towards different DNA structures have been carried out and achieved tremendous advancements. In this article, the main achievements of BBR derivatives in polymorphic DNA structures binding researches in recent 20 years were reviewed. The structural modification strategy of BBR, the DNA binding effects of its derivatives, and the structure activity relationship (SAR) analysis have also been discussed.

## Introduction

**FIGURE 1:**
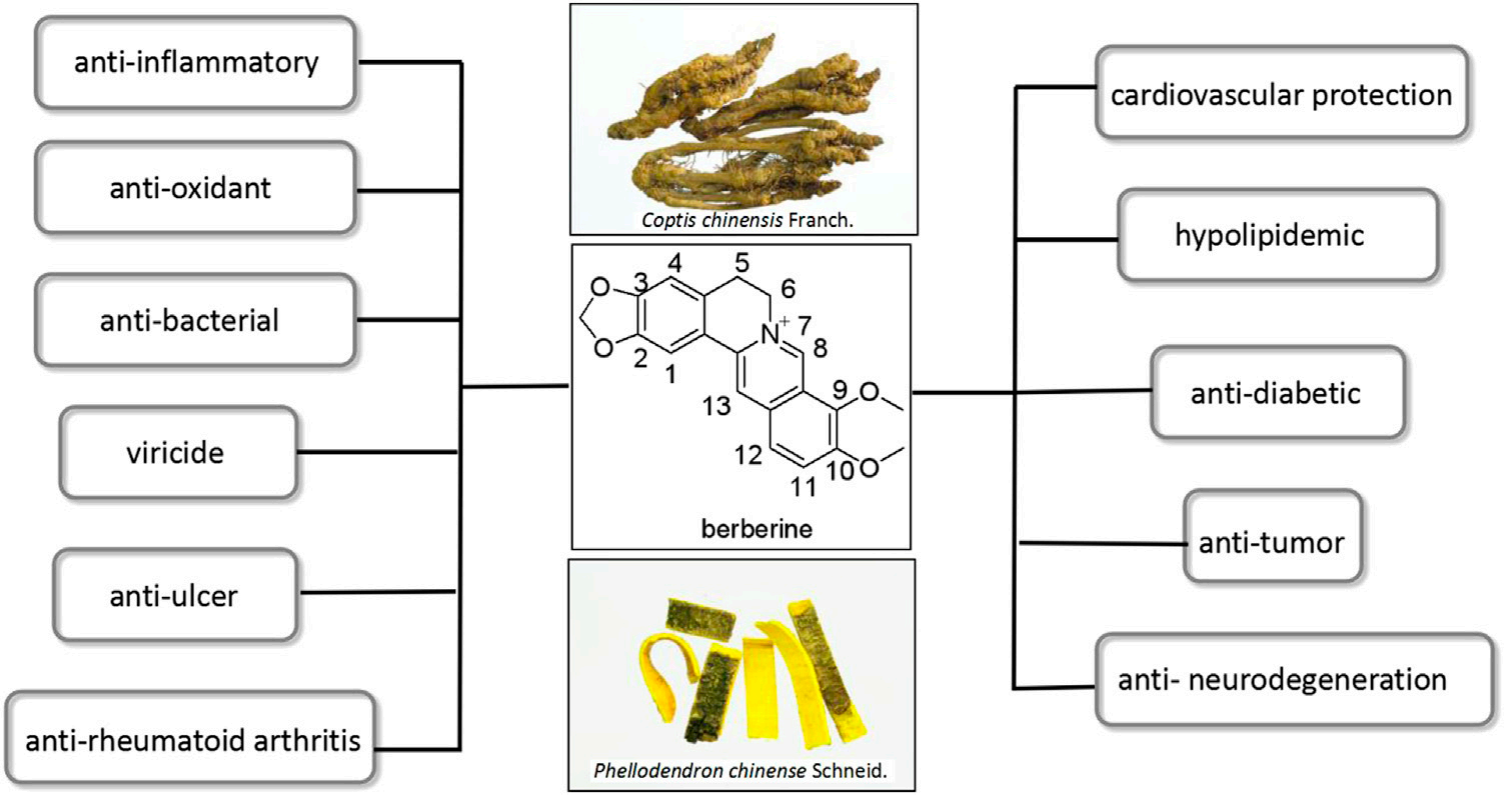
Pharmacological activities of BBR.

Berberine (BBR, Figure 1) is the most important quaternary benzylisoquinoline alkaloid widely distributed in several botanical families, such as Ranunculaceae (Coptis chinensis), Rutaceae (Phellodendron amurense), and *berberiaceae* (Berberis thunbergii), and so on (Imanshahidi and Hosseinzadeh, 2008; Bhadra and Kumar, 2011). It has been used in Traditional Chinese Medical systems in China and Ayurvedic system in India for a very long time (Gaba et al., 2021; Xu et al., 2021). Modern pharmacological studies have proved that BBR has abundant biological activities including anti-inflammatory (Bai et al., 2020; Ma et al., 2020; Fu et al., 2021), anti-oxidant (Eissa et al., 2018; Seth et al., 2021), anti-bacterial (Wang et al., 2009; Avci et al., 2018; Tong et al., 2021), viricide (Hayashi et al., 2007; Cecil et al., 2011; Hung et al., 2019), cardiovascular protection (Salehi and Filtz, 2011; Li et al., 2014; Wang L. et al., 2020), hypolipidemic (Hu et al., 2012; Zhou et al., 2014), anti-diabetic (Lee et al., 2006; Ye et al., 2016; Di et al., 2021), anti-tumor (Liu et al., 2017; Zhang et al., 2020; Ren et al., 2021), anti-ulcer (Li et al., 2020; Xiong et al., 2021), anti-neurodegeneration (Liang et al., 2017; Hussien et al., 2018; Živančević et al., 2022), and anti-rheumatoid arthritis (Huang et al., 2021; Li et al., 2022) (Figure 1).

**FIGURE 2:**
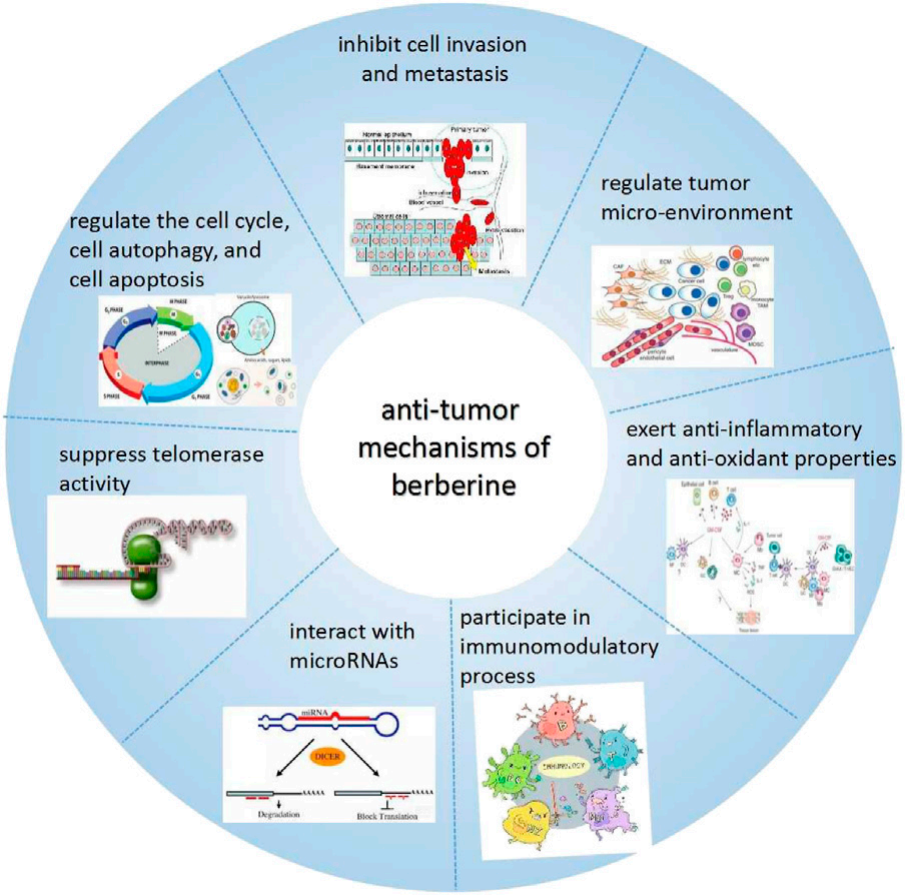
The anti-tumor mechanisms of BBR.

In recent years, the anti-tumor effects of BBR have attracted more and more attention of the researchers. BBR can constrain the growth of cancer cells through diverse mechanisms (Guamán Ortiz et al., 2014; Liu et al., 2017; Zhang et al., 2020; Ren et al., 2021), such as cell cycle regulation, autophagy, and inducing apoptosis, repressing cell invasion and metastasis, regulating tumor micro-environment, exerting anti-inflammatory and-oxidant effects, immunomodulation, interacting with micro ribonucleic acids (microRNAs) and suppressing telomerase activity, etc (Wang Y. et al., 2020) (Figure 2).

Deoxyribonucleic acid (DNA) is a right-handed double-stranded helical macro-molecule made up of two polydeoxyribonucleotide chains (Watson and Crick, 1953). DNA carries genetic information, either mutation or unusual rearrangement of DNA can affect gene expression and other biological processes (Khan et al., 2012; Sjakste et al., 2020). The canonical right-handed DNA (B-DNA) has the capacity to transform into its polymorphs that are often co-localized with mutation sites which are implicated in genetic instability-related diseases such as tumor. More than a dozen of polymorphic DNA structures has been reported, including A-DNA, Z-DNA, H^L^-DNA, protonated DNA, triplex DNA, and quadruplex DNA, etc (del Mundo et al., 2019; Maiti and Kumar, 2007a).

The special planar tetracyclic system of BBR makes it a good candidate for binding with different DNA structures. It exhibited strong affinity to B-DNA with AT base pair preference by partial intercalation in which the planar isoquinoline moiety intercalated between the base pairs (Mazzini et al., 2003; Chen et al., 2004). It can also bind to the left-handed H^L^-DNA. The interaction of BBR with H^L^-DNA resulted in opposite sign swift and magnitude compared to B-DNA (Kumar et al., 2003). BBR could be used as a sensitive probe to detect the alteration of structural handedness due to the change in DNA on protonation. In addition, BBR showed stronger binding affinity towards triplex T×AT DNA compared with AT duplex (Ren and Chaires, 1999). This process stabilized the Hoogsteen strand of triplex structures without affecting the Watson-Crick strands, that leads to its use in triplex DNA-targeted therapies (Das et al., 2003). BBR showed inhibitory activity on telomerase elongation and bond to human parallel G-quadruplex DNA (G4 DNA) through stacking interaction on the terminal G-tetrad of quadruplex (Naasani et al., 1999; Parkinson et al., 2002). In an examination to detect the interactions of BBR with various G4 DNA structures and duplex DNA, it was found that BBR showed selectivity towards G4 DNA structures in contrast to duplex DNA, which verified the dominant role of aromatic moiety in BBR for G4 DNA binding. Moreover, BBR is selective in inducing intermolecular G4 DNA compared to intramolecular ones (Franceschin et al., 2006). The differential binding affinity of BBR with various polymorphic DNA structures may bring about a new avenue for future rational design of BBR derivatives with efficient and selective anti-tumor activities (Maiti and Kumar, 2007b).

**FIGURE 3:**
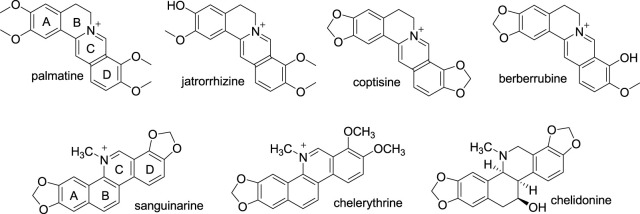
The structures of representatives of PBs, and benzo[c] phenanthridines.

Protoberberines (PBs) are a group of alkaloids with the same 5,6-dihydrodibenzo[a,g] quinolizinium (C_17_H_14_N^+^) skeleton (Grycová et al., 2007). BBR is the most widely distributed PB. Besides BBR, palmatine (PMT), jatrorrhizine (JAT), coptisine (COP), and berberrubine (BBRB) are other representatives of PBs (Figure 3). They share a common skeleton, only with substituents on position 2, 3, 9, 10 different. So, they show close similarities in their biological properties (Basu and Kumar, 2018). A study aiming at clarifying the SAR of different PBs with ds DNA of different sequences revealed that PMT, and JAT, COP possessed better binding affinities towards the three test ds DNAs than BBR, and BBRB by electrospray ionization mass (ESI-MS) spectrometric method. While, BBR showed higher binding affinity than PMT by fluorescence spectrometric method. Combining the results of the two methods indicated that the slight structural differences of these five alkaloids had no significant impact on their activities towards ds DNA (Chen et al., 2005a). Like BBR, PMT also showed AT base pair preference by mechanism of intercalation (Bhadra et al., 2007). In addition to binding to ds DNAs, PMT also binds to G4 DNA and RNA. The affinities of PMT towards G4 DNA and different RNAs are comparable to BBR, but a little weaker (Giri et al., 2006; Islam et al., 2008; Xiong et al., 2015a; Kumar and Barthwal, 2018). Compared with PBs, benzo[c]phenanthridines also belong to isoquinoline alkaloids. They possess the similar quaternary nitrogen, polycyclic and planar structure with PBs and showed obvious binding affinities towards natural and synthetic DNAs. Sanguinarine (SG), chelerythrine (CHE), and chelidonine (CHEN) are the most common members of the benzo[c]phenanthridine family (Figure 3). SG and CHE exhibited DNA damage and cytotoxicity in both primary mouse spleen cells and mouse lymphocytic leukemic cells (L1210 cells) in a dose-dependent manner. While CHEN did not show a significant damage DNA or cytotoxicity in both cell types, but it can also arrest the growth of L1210 cells (Kaminskyy et al., 2008). The results of a ds DNA binding experiment indicated that SG can intercalate in ds DNA molecule with GC base pair preference. While CHEN interacts with it weakly (Bashmakova et al., 2008). The fact that the planarity of CHEN is not as good as SG may explain their difference in DNA binding mode, and DNA damage and cytotoxicity in mouse cells. Another ct DNA binding assay manifested that the interactions of CHE and SG with ct DNA are mainly via intercalation. SG intercalates into the DNA base pair with its C and D rings. Because the existence of two methoxyl moieties on its D ring destroys its planarity, CHE intercalates to ct DNA with its A and B rings. The binding affinity of CHE to ct DNA is 3-fold weaker than that of SG (Li et al., 2012). Additionally, SG also exhibited excellent affinities to intercalate into protonated DNA and bind to single stranded RNA (Giri and Kumar, 2007; Sen et al., 2020). The structures of various PBs, and benzo[c]phenanthridines with DNA binding effects can provide direction for the development of BBR derivatives.

Although, BBR possesses binding effects towards polymorphic DNA structures, the affinity is modest and its bioavailability is undesirable due to its poor lipophilicity and fast metabolism, which hinders its application in clinical (Liu et al., 2016). Structural modifications of natural products are always effective means to improve activities and reduce toxicities. BBR, as a very promising pharmacophore (Singh et al., 2021), has been optimized in various ways to improve its binding effects with polymorphic DNA structures.

The modifications of BBR have been reviewed by Singh and Mahajan (2012), Jin et al. (2016), Zou et al. (2017), and Gaba et al. (2021). Singh’s and Jin’s reviews compiled the patents related with BBR derivatives before 2012, and 2016, and mainly discussed their pharmacological applications (Singh and Mahajan, 2012; Jin et al., 2016). Zou’s review focused on the anti-inflammatory and anti-tumor advances of BBR derivatives in digestive system (Zou et al., 2017). Gaba’s review covered the development of BBR derivatives from 2016 to 2020, emphasizing on their chemical diversities, biological activities, structure activity relationships (SARs), molecular modelling and mechanistic studies (Gaba et al., 2021). Each review described the advances of BBR derivatives from different angles. In this review, the structural modifications of BBR, the double-stranded DNA (ds DNA) and G4 DNA binding effects of its derivatives, and the corresponding SAR will be summarized, which will make a good supplement to previous reviews. And it will be of great help on anti-tumor drug design and development of BBR as the lead compound towards polymorphic DNA structures bindings.

## Materials and methods

The information of BBR, polymorphic DNAs, modifications of BBR and the ds DNA and G4 DNA binding effects of BBR derivatives was collected by systematic survey of literature. All the reference materials were retrieved from scientific databases, such as Pubmed, Elsevier, Web of Science, Springer, Willey, and CNKI. The keywords used as search terms included “berberine”, “berberine’s pharmaceutical effects”, or “berberine’s activities”, “DNA”, “polymorphic DNAs”, “modification of berberine”, or “berberine derivatives”, “berberine derivatives’ DNA binding effects” in all the fields. The results were then cross-referenced to generate maximum number of articles available.

### Progress in the modifications of BBR for polymorphic DNA structures bindings

#### The bindings of ds DNA

**FIGURE 4:**
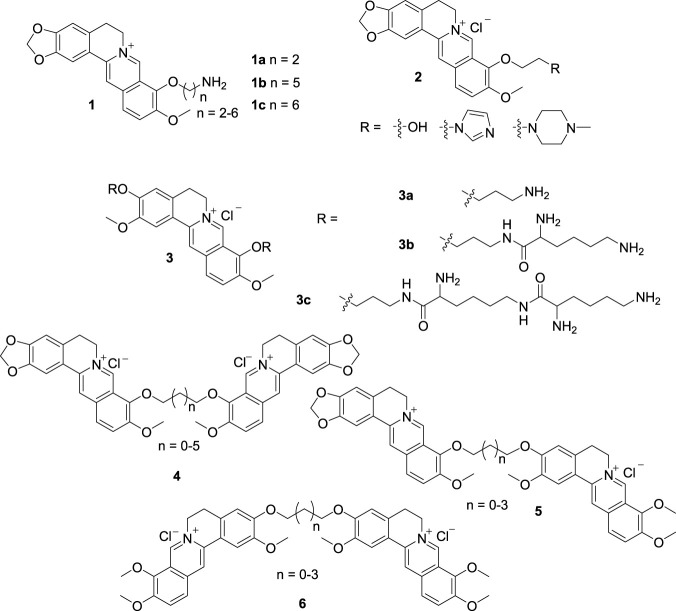
The structures of compounds **1**-**6**.

DNA variation is associated with many diseases, such as tumor, virus infection, and so on (Blackledge and Melander, 2013). There is an increasing interest in the development of DNA-binding agents that can be applied to explore the structure and function of DNA, to elucidate the action mechanism of anti-tumor and anti-virus drugs and to develop new chemotherapeutic agents (Chen et al., 2005b; Chaires, 2015). BBR is an attractive lead compound for development of potential DNA-binding drugs in view of its ubiquitous pharmacological effects and unique structure. In 2005, Chen and Jiang et al. reported four 9-monomodified BBR derivatives (**1a**, and **2**, Figure 4) for DNA-binding evaluation (Pang et al., 2005). The functional groups introduced into BBR are frequently used in the design of DNA-binders. The results of biophysical and biochemical experiments indicated that the four BBR derivatives, especially **1a** with a primary amino terminal, strongly bond with calf thymus DNA (ct DNA), presumably via an intercalation mechanism.

Based on the fact that position 9 of BBR decorated with a primary amino on the endpoint can enhance the DNA-binding activity, as well as the high DNA-binding affinities displayed by polyamines, three new PB derivatives bearing two to six primary amino groups at positions 3 and 9 (**3**, Figure 4) were synthesized and evaluated for their binding affinities towards ct DNA by Chen’s group in 2007 (Pang et al., 2007). The results indicated that **3** possessed quite high DNA-binding affinities attributed to the multivalent interactions between the polyamino groups and DNA. While, **3c** bearing six amino groups was only slightly more effective in DNA binding than **3b** bearing four amino groups. These PB derivatives are exploitable as effective DNA-binding agents.

From 2005 to 2006, Chen synthesized a series of alkyl diether linked BBR homodimers (**4**, Figure 4), BBR-JAT heterodimers (**5**, Figure 4) and JAT homodimers (**6**, Figure 4) (Chen et al., 2005b; Long et al., 2006; Qin et al., 2006). Both BBR and JAT are belonging to PB alkaloids, which exhibit extensive pharmacological activities. The interactions of **4** with ct DNA and two 10-mermbered ds DNA [d(AAGAATTCTT)_2_, d(AAGCATGCTT)_2_] and one 12-mermbered ds DNA [d(TAAGAATTCTTA)_2_] indicated that they possessed higher binding affinities towards the four DNAs than their monomeric BBR. To 10- and 12-mermbered ds DNAs, **4** showed a prominent SAR related to the length of the alkyl linkers. The dimer linked by a propyl chain exhibited the highest affinity. But no obvious SAR was observed with ct DNA, because ct DNA is a mixture of different ds DNA chains. In addition, **4** showed higher binding affinities with 12-mermbered ds DNA than with 10-mermbered ds DNA, because they can occupy a greater number of base pairs in 12-mermbered ds DNA. The DNA-binding mode of BBR and **4** was proposed to be intercalating. The quaternary ammonium cation and planar structure of **4** played key roles in the DNA-binding process (Chen et al., 2005b; Qin et al., 2006). Based on the results of **4**, compounds **5** and **6** were synthesized (Long et al., 2006). **5** and **6** showed much higher DNA-binding affinities than their monomeric components similar with **4**. For **6**, the best linker was ethyl chain, which is different from **4**. Whereas **5** possessed comparable binding abilities, which suggested that they averaged the effects of BBR homodimers and JAT homodimers. It meant that the binding affinities of PB dimers can be modulated by varying the length of the linkers. For the PB dimers with the same linkers, BBR-containing dimers showed higher binding affinities than JAT homodimers. Because PMT, the parent compound of JAT, has comparable DNA-binding affinity with BBR (Qin et al., 2007a), it is supposed that attaching positions have significant impacts on the DNA-binding affinities of PB dimers.

Topoisomerases are DNA processing enzymes that relieve supercoiling (torsional strain) in DNA (Castelli et al., 2012). There are two types of topoisomerases, Top I and Top II. Top I catalyzes the conversion of the DNA topology by introducing single-strand breaks into the DNA molecule during basic cellular processes such as DNA replication, transcription, recombination, repair and chromatin remodeling (Parchment and Pessina, 1998; Giovanni et al., 2008). Top II is able to cleave ds DNA and support more complex interconversions of the DNA topology by an ATP- dependent strand passage mode in transcription, replication, and segregation of chromosomes processes during cell division (Sutormin et al., 2021). Due to their crucial roles in replication and transcription, Top I and Top II are attractive clinical molecular targets for cancer therapy by camptothecin series and doxorubicin series of antineoplastics agents, respectively. In order to reveal the relationship between drug-DNA interaction and Top I poisoning, Jiang et al. further evaluated the Top I inhibitory activities of natural PBs, monomodified BBRs (**2**), BBR homodimers (**4**), JAT homodimers (**6**), and BBR-JAT heterodimers **5**) (Qin et al., 2007b). The results revealed that *P*Bs and **2** showed only weak activities, while the PB dimers showed stronger Top I inhibition potency than their natural congeners or monomodified BBRs, which was consistent with the SAR result discovered in previous DNA-binding studies. The mechanism of Top I inhibition by dimeric PBs was concentration-dependent. At low concentration, they inhibit Top I by stablization of the enzyme-mediated DNA “cleavable complex” as camptothecin does. At high concentration, obvious inhibition of the relaxation activity of Top I is observed, probably due to their high binding affinities towards plasmid DNA. The findings of Chen and Jiang et al. also suggests that the synthesis of dimeric alkaloid analogues is a useful tool in rational drug design for the cooperative interactions of the two subunits targets.

In 2011, Kumar’s group synthesized five 9-*O*-amino alkyl BBR derivatives (**1**, Figure 4) and determined their interactions with ct DNA (Islam et al., 2011a). All the derivatives dramatically enhanced the fluorescence emission and remarkably enhanced the DNA binding affinities compared with BBR. The binding affinity was directly dependent on the alkyl chain length. **1b** and **1c** with long alkyl chains exhibited remarkable changes in the spectral and binding properties.

Compared with the relatively simple ds DNA structure, RNA molecules have diverse and complex structures. The plentiful roles of RNA playing in the progression of many diseases, particularly cancers, have led to a growing interest in exploiting RNA as a cellular target for therapeutic drugs (Tucker and Breaker, 2005). Studies on the interaction of small molecules with polyadenylic acid [poly (A)] have been reported in recent years (Hyun et al., 2006). In view of these, Kumar evaluated the interactions of **1** with poly(A) (Islam et al., 2011b). The results showed that all the compounds including BBR induced cooperatively self-structure formation in poly(A). The length of the alkyl chain had a significant influence on the formation of self-structure. The reasons may be that the flexibility of the alkyl chain confers a better geometry probability for the 9-terminal amino group interacting with poly(A) base pairs and phosphates. It is pertinent to mention that in the previous study the DNA binding affinities of these analogues have been found to be enhanced by about thirty times. This study further confirmed the importance of 9-*O*-substitution in BBR and the role of flexible and long alkyl chain for stronger nucleic acid binding.

**FIGURE 5:**
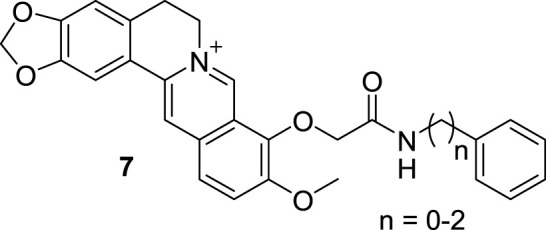
The structures of compounds **7**.

Then Kumar synthesized another three 9-*O*-*N*-aryl/aryl-alkyl amino carbonyl methyl BBR derivatives (**7**, Figure 5) and evaluated their DNA binding properties (Basu et al., 2012). The results revealed that **7** retained the DNA binding mode of intercalation and showed remarkable enhanced binding affinities and thermal stabilization of DNA. Although the binding affinities of the derivatives were dependent on the lengths of the side chains, further chain elongation gradually reduced the binding affinity. Their thermal stabilization effects of DNA may be ascribed to the side chains, for their participation in hydrogen bonds formed with either the base pairs or the phosphates of DNA. All the derivatives bounded to DNA non-cooperatively through multiple weak non-covalent interactions, different form the parent alkaloid BBR.

**FIGURE 6:**
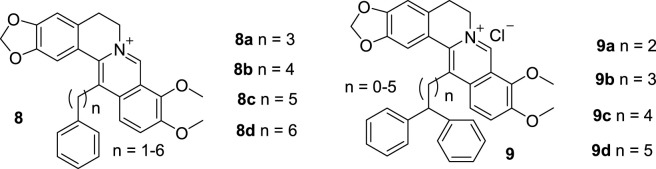
The structures of compounds **8** and **9**.

While studying 9-substituted BBR derivatives, Kumar also synthesized a series of 13-substituted BBR derivatives. Six BBR derivatives with alkyl chains of various length and a terminal phenyl group at position 13 (**8**, Figure 6) were synthesized and investigated for their binding effects with ct DNA and nucleic acid triplex (Bhowmik et al., 2012; 2014a). Nucleic acid triple helices are oligo-nucleotides containing natural units, which can tightly bind to the major grooves of complementary DNA and RNA duplexes and were used to treat a wide range of diseases including tumor (Jain et al., 2008). Because they are not stable under physiological conditions, it is necessary to take advantage of small molecules to bind with them so as to improve their stability (Escudé et al., 2001). In the ct DNA binding study, all the derivatives bond to ct DNA non-cooperatively. The binding affinities increased until the chain length up to three -CH_2_- unites. The DNA stabilization effects were achieved by intercalating. The complexation effects were dominated by nonpolyelectrolytic forces, while polyelectrolytic forces contributed only a quarter to the total free energy (Bhowmik et al., 2012). In the nucleic acid triplex binding study, the similar results were found as ct DNA binding study. **8** bond non-cooperatively to the RNA and DNA triplex by intercalation. The affinity enhanced up along with the linker length of the substituent increasing, until the length reached to four -CH_2_- unites. They can change the conformations of the triplexes and enhance their stability. Moreover, energetics of the interaction revealed that the binding was more entropy driven, as the alkyl chain length increased (Bhowmik et al., 2014a).

In 2014, Kumar synthesized six 13-diphenylalkyl BBR derivatives (**9**, Figure 6) and evaluated their DNA binding affinities (Bhowmik et al., 2014b). The biophysical evaluation results demonstrated that all the compounds showed improved binding abilities towards one natural and two artificial ds DNAs. The binding affinities increased along with the lengthening of the linker alkyl groups, until the length reached three -CH_2_- unites. In addition, **9** displayed great preference to AT abundant sequences compared with GC abundant sequences.

These results verified that substitution of position 13 is also a good avenue to enhancing the DNA binding ability of BBR.

#### The bindings of G4 DNA

G4 DNA is a type of quadruple helix structure formed by a continuous guanine-rich DNA sequence (Luedtke, 2009). It is involved in several physiological processes, such as regulation of DNA replication, transcription of disease-related genes (c-MYC, BCL-2, KRAS, c-KIT, etc), telomere maintenance, and epigenetic regulation. It has been considered as a novel and promising target for anti-tumor drug design (Teng et al., 2021).

**FIGURE 7:**
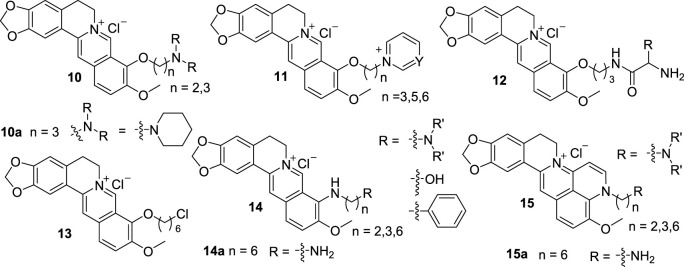
The structures of compounds **10**–**15**.

From 2007 to 2015, Huang’s group reported a series of 9-substituted, and 8, 9-cyclized BBR derivatives (**10**–**15**, Figure 7), and investigated the interactions between these derivatives and G4 DNA (Zhang et al., 2007; Ma et al., 2008; Ma et al., 2009; Ma et al., 2011; Ma and Huang, 2012; Xiong et al., 2015b). Compounds **10** are 9-*O*-substituted BBR derivatives with different amino alkyl side chains (Zhang et al., 2007). They exhibited stronger binding affinities with G4 DNA and higher inhibitory activities for telomerase than BBR. The activities of compounds with three -CH_2_- unites side chains are stronger than those with two -CH_2_- unites side chains. The substitutions of position 9 with amino terminals are beneficial to the activities. **10a** with a terminal piperidine group displayed the best activity. Compared with **10**, compounds **11** contain aza-aromatic terminal groups in their structures (Ma et al., 2009). **11** owning positive charged aza-aromatic terminal groups had high binding affinities and superior selectivity in biophysical evaluation, polymerase chain reaction (PCR) termination experiment, and molecular docking studies because of the possible multiple interactions of them with G-quartet, grooves and loops of G4 DNA. When the linker length is more than three -CH_2_- unites, it has subtle influence on activity. From the results of **10** and **11**, it can be deduced that the length of the linker and the alkalinity of the terminal group on position 9 contribute to the interactions between BBR derivatives and G4 DNA.

According to the structures of **10** and **11**, compounds **12** with three kinds of basic amino acid residues were designed and synthesized (Ma and Huang, 2012). They showed better stabilization potency and selectivity towards G4 DNA than BBR. And they can effectively prevent DNA amplification. From the SAR analysis, it can be concluded that the activity will be enhanced with the increase of the amino group number of the side chain, the alkalinity of the terminal group and the length of the linker, which is consistent with the results of **10** and **11**.

Compound **13** was found by virtual screening campaign on Huang’s in-house library of 9-substituted BBR derivatives with long side chains (Xiong et al., 2015b). *In vitro* and *in vivo* studies indicated that it can bind to G4 DNA and cause the dissociation of shelterin proteins, which lead to cell cycle arrest, DNA damage, and cell senescence in cancer cells.

When developing 9-*O*-substituted BBR derivatives, Huang also designed and synthesized a series of 9-*N*-substituted BBR derivatives (**14**). The terminal groups on position 9 of **14** are mainly nitrogen-containing groups, additionally, hydroxyl and phenyl groups (Ma et al., 2008). The interactions of **14** with G4 DNA in the promoter region of the c-MYC indicated that **14** could selectively stabilize the parallel G-quadruplet structure formation in c-MYC DNA, thus lead to down-regulation of transcription of c-MYC in HL60 cell line. **14a** with a 1,6-diaminohexyl side chain on position 9 showed the best inhibitory activity and selectivity. Although the activities of **14** were not higher than those of the previously synthesized compounds, they increased the diversity of BBR derivatives.

Because expanding the size of aromatic planar surface of the target compound might enhance its binding affinity and selectivity towards G4 DNA, a series of quinoline-benzo-[5,6]-dihydroisoquindolium compounds (**15**) were designed and synthesized by cyclization of position 8 and 9-amino groups in **14** (Ma et al., 2011). Biophysical and biochemical evaluation demonstrated that the introduce of pyridine ring into BBR scaffold improved the binding ability and selectivity towards c-MYC G4 DNA. Furthermore, **15a** with the same side chain as **14a** showed excellent inhibitory effect on the transcription of c-MYC in HL60 cell line but slightly affect normal ECV-304 cell, which was consistent with the behavior of an effective G4 DNA ligand targeting c-MYC oncogene. The results also indicated that expanding of central aromatic moiety of BBR was another practicable way to improve the stabilizing potency and selectivity of BBR derivatives towards G4 DNA over ds DNA.

**FIGURE 8:**
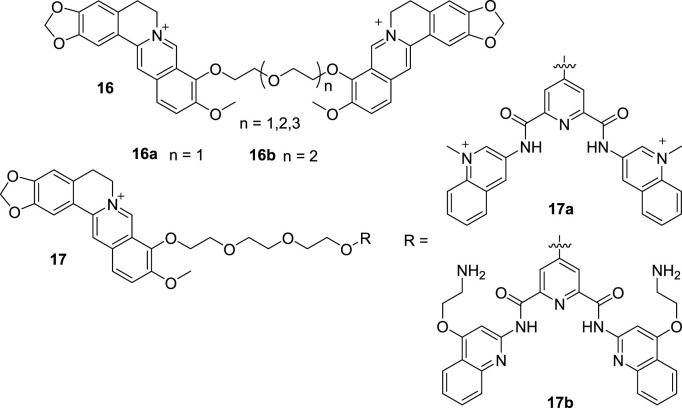
The structures of compounds **16** and **17**.

Although monomeric G4 DNA is common, there are still some multimeric G4 DNAs, which are also associated with some cancers (Frasson et al., 2022). Subsequently, improved dimeric or multimeric G4 DNA binders have been developed to find potential anti-tumor drugs. From 2016 to 2017, Zhou and Chen et al. reported three polyether-tethered dimeric BBR derivatives (**16**, Figure 8) (Zhou et al., 2016; Li et al., 2017). After the binding affinity, selectivity and thermal stabilization detections towards dimeric and monomeric G4 DNA, **16a** with the shortest polyether linker showed the best activity towards mixed-type dimeric G4 DNA over anti-parallel dimeric G4 DNA and three monomeric G4 DNA via end stacking and external binding modes. **16b** with modest polyether linker showed highest binding affinity to anti-parallel dimeric G4 DNA, but it did not show discrimination towards mixed-type and anti-parallel dimeric G4 DNA. The polyether linkers in the compounds played important roles in regulating the binding affinity and selectivity towards mixed-type dimeric G4 DNA. Bisquinolinium scaffold has been proved to be a G4 DNA binder in Zhou’s previous study (Liao et al., 2018). In 2020, Zhou’s group synthesized another two BBR-bisquinolinium conjugates (**17**, Figure 8) (Liao et al., 2020). The results of the DNA binding affinity evaluation indicated that compounds **17** were selective, sensitive and fluorescence responsible to distinguish human telomeric dimeric G4 DNA from other types of G4 DNA and ds DNA by binding to two adjacent G4 DNA units. They also possessed strong telomerase inhibitory activities and toxicity towards tumor cell lines. Microscopy experiments demonstrated that **17b** entered the nucleoli and targeted G4 DNA in organelles. These results suggest that the combination of an efficient optical tag and a well-known G4 DNA binder is an effective approach to identify new imaging agents for dimeric G4 DNA. From Zhou’s research, it can be concluded that highly selective probes for dimeric and/or multimeric G4 DNA may help to understand the structures and functions of G4 DNA and provide useful guidance for the rational design of G4 DNA-targeting anti-tumor drugs.

Telomerase is an RNA-dependent DNA polymerase, which can extend the telomeres of chromosomes and counteract progressive telomeres shortening during cellular replication. High telomerase activity is detected in about 90% of human tumors. An important characteristic of tumor cells is uncontrollable proliferation, which is mainly achieved by telomerase-mediated telomere maintenance in the majority of advanced tumors (Buseman et al., 2012). Telomerase inhibitors are able to disrupt the replicative capacity of telomerase-positive cancer cells and could be used as selective anti-tumor therapeutic agents. G4 DNA stabilizing is a good way to inhibit telomerase activity. This sequestering mode can interfere the access of the telomerase to its substrate and, therefore, the elongation of the telomeres (Calvo and Wasserman, 2016). Platinum-based complexes are kinds of famous anti-tumor drugs, which could also be used as excellent G4 DNA stabilizers (Bai et al., 2017). They can induce high degrees of G4 DNA stabilization and inhibit telomerase activity (Wei et al., 2013).

**FIGURE 9:**
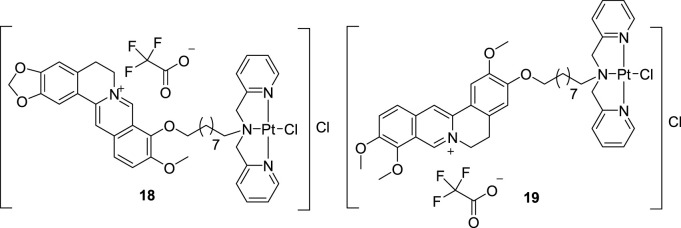
The structures of compounds **18** and **19**.

In 2019, Qin et al. synthesized a BBR-Pt (II) complex (**18**, Figure 9) and a JAT-Pt (II) complex (**19**, Figure 9) and explored their inhibitory activities towards telomerase (Qin et al., 2019). *In vitro* and *in vivo* cytotoxicity results suggested that these two compounds showed good anti-proliferative activities towards human bladder T-24 tumor cells, especially **19**. They can induce cancer cell apoptosis via targeting telomerase, along with inducing mitochondrial dysfunction, damaging telomere DNA and arresting cell cycle. These two complexes showed low systemic toxicities and could be used as novel platinum-based anti-tumor drug candidates.

Kumar’s group dedicated much efforts in the development of DNA binding agents. In 2015, they conducted a telomeric G4 DNA sequence binding experiment with two most potent compounds (**1c**, Figure 4, **8a**, Figure 6) in the previous DNA binding experiment (Islam et al., 2011a; Bhowmik et al., 2012; Bhowmik et al., 2015). **1c** is a 9-substituted BBR derivative and **8a** is a 13-substituted BBR derivative. Both of them exhibited stronger binding affinity than BBR. And the binding mode was non-cooperative binding through stacking interaction. A series of biophysical assays verified that the two compounds could stabilize the structure of G4 DNA, and **1c** showed better activity than **8a**. Compounds with DNA binding activities can also be used for the development of G4 DNA binding agents.

**FIGURE 10:**
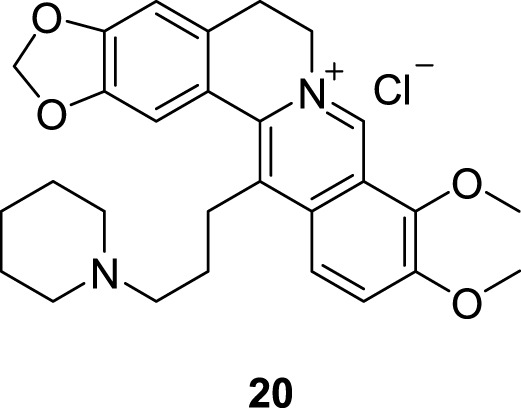
The structure of compound **20**.

In 2006, Franceschin in Neidle’s group synthesized 13-[3-(1-piperidino) propyl] BBR hydrochloride (**20**, Figure 10), which showed good selectivity for G4 DNA compared to ds DNA. Molecular modelling studies indicated that the aromatic moiety stacked on the terminal G-quartet of G4 DNA, and the 3-(1-piperidino) propyl side chain can interact with one of the four grooves of G4 DNA (Franceschin et al., 2006). The aromatic planar moiety of BBR and the side chain on position 13 are indispensable for binding to G4 DNA.

**FIGURE 11:**
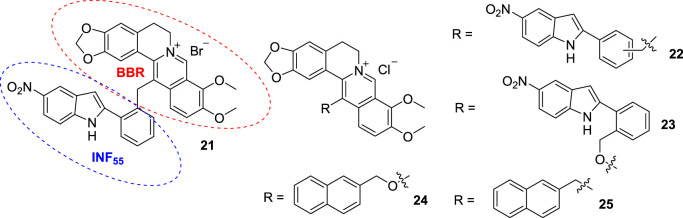
The structures of compounds **21**–**25**.

In 2006, Bremner’s group synthesized a new compound (**21**, Figure 11) by conjugating BBR to a multi-drug resistance pump inhibitor, INF_55_, for anti-microbial research (Ball et al., 2006). In 2007, Beck’s group took **21** as a candidate for G4 DNA binding and determined the binding affinity of it towards G4 DNA and ds DNA (Gornall et al., 2007). The results showed that the binding affinity of **21** for G4 DNA is modest. However, its preference for G4 DNA over ds DNA is obvious. In 2010, Beck assessed the binding affinities of compounds **22** (Figure 11), reported by Bremner (Bremner and Kelso, 2010), compounds **23** and **24** (Figure 11), reported by Samosorn’s group (Samosorn et al., 2009) and the newly synthesized compound **25** (Figure 11) to inter- and intra-molecular G4 DNA (Gornall et al., 2010). Compounds **22** are *meta* and *para* isomers of **21** with chlorine as the negative ion part. The results manifested that all the compounds, except for **24**, can stabilize the quadruplet structure of G4 DNA and perform selectivity for G4 DNA over ds DNA. The reason may be that the molecular structure of most compounds are not planar, they are unlikely to bind to ds DNA as classic minor groove binding or intercalating ligands. These 13-substituted derivatives may serve as a new class of G4 DNA-selective ligands.

**FIGURE 12:**
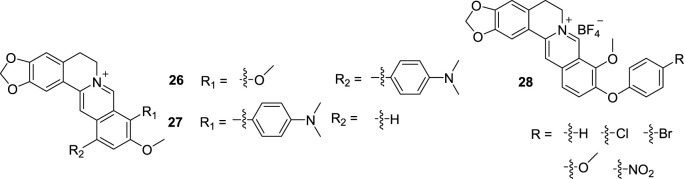
The structures of compounds **26–28**.

In 2021, Ihmels’s group successfully synthesized 9-dimethylaminophenyl BBR and 12-dimethylaminophenyl BBR (**26** and **27**, Figure 12) (Wickhorst and Ihmels, 2021). They bond to G4 DNAs with high affinities, both at neutral conditions and at pH 5. While the binding constant towards ct DNA is lower at pH 5 than under neutral conditions. An increase of emission intensity upon association with G4 DNAs at pH 5 was also observed. That is to say, only when the simultaneous detection of G4 DNA and lower pH values are both met, compounds will light up as fluorescent probes. This property may be applied for selective fluorimetric detection of G4 DNA in cancer cells, which often provide a medium with slightly lower pH than healthy cells.

At the same time, Ihmels synthesized a series of 10-*O*-aryl-substituted BBR derivatives (**28**, Figure 12) and studied the DNA-binding properties of them (Wickhorst et al., 2021). The derivatives showed moderate affinity towards G4 DNA and ct DNA and exhibited fluorescence light-up effects upon complexation to both DNA forms, with slightly higher intensity to G4 DNA. It was also revealed that **28** bind to ds DNA by intercalation and G4 DNA by terminal *π* stacking. Although the derivation of position 10 on BBR is rare, it is a method worth considering to develop new G4 DNA stabilizer.

#### SAR analysis

**FIGURE 13:**
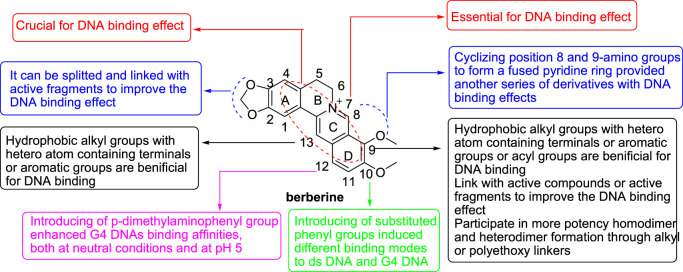
SAR of BBR derivatives towards ds DNA and G4 DNA.

Taken together, the SAR of BBR derivatives towards ds DNA and G4 DNA could be concluded (Figure 13). The unique aromatic planar surface combined by the four rings of BBR and its derivatives plays a vital role in their DNA binding effects, because it can intercalate into the base pairs of different DNA structures. Meanwhile, the quaternary ammonium nitrogen atom is also very important. The reason may be that the cation can form a strong ionic interaction with the target. The structural modifications of BBR to improve its DNA binding effects are mainly focused on positions 9, and 13. In general, hydrophobic alkyl groups with hetero atom containing terminals or aromatic groups or acyl groups introduced to these positions are favorable to improve the lipophilicity of BBR, balance its lipid water partition coefficient and increase the bioavailability of BBR. In addition, the introduced hydrophobic groups can form hydrophobic bonds or aromatic ring interactions with the targets and the hetero atoms in the terminal groups or acyl groups can from hydrogen bonds with the targets. SAR analysis showed that the length of the side chain significantly influence on the DNA binding affinities of BBR derivatives. Beyond that, expanding the size of the central aromatic moiety of BBR, combining BBR with some active compounds or active fragments, homodimerizing of BBR or heterodimerizing of BBR with other PB, are also common structural modification methods to enhance its DNA binding affinity and selectivity, and DNA stabilizing effect. Introducing of substituted phenyl groups to position 10 did not improve the activity, but can induce different binding modes towards ds DNA and G4 DNA. Introducing of p-dimethylaminophenyl group to position 12 enhanced G4 DNAs binding affinities, both at neutral conditions and at pH5.

## Conclusions and perspective

In conclusion, the DNA binding researches of modified BBR derivatives mainly focused on ds DNA and G4 DNA, in addition to nucleic acid triplex and single stranded RNA. The unique aromatic planar surface combined by four rings of BBR and its derivatives plays a vital role in their DNA binding effects. The quaternary ammonium nitrogen atom is also a key component. The structural modifications of BBR to improve its DNA binding effect are mainly focused on positions 9, and 13. SAR analysis showed that the length of the side chain on positions 9, and 13 exerted significant influence on the DNA binding affinities of BBR derivatives. Positions 2, 3, 10 and 12 were seldom modified (Table 1).

**TABLE 1:** The main modification strategies of BBR towards polymorphic DNAs.

MainTargets	Modification position	Introduced groups
ds DNA	9-*O-*	Aminoalkyl, Hydroxyalkyl, Alkyl tethered PBs, Aminocarbonyl methyl
	13-	Aralkyl
G4 DNA	9-	Dimethylamino phenyl
	9-*O-*	Aminoalkyl, Amino acid amide alkyl, Chloroalkyl, Polyether tethered BBR, Polyether tethered bisquinolinium scaffold, Alkylamine tethered Pt-complex
	9-*N-*	Aminoalkyl, Phenylalkyl, Hydroxyalkyl
	8 and 9	Fused pyridine
	10-	Substituted phenyl
	12-	Dimethylamino phenyl
	13-	Aminoalkyl, Aryl methyl, Aryloxy methyl
Triplex DNA	13-	Phenylalkyl
RNA	9-*O-*	Aminoalkyl

While, the BBR derivatives synthesized by different groups overlapped sometimes and the modification strategies were slightly the same, which is not beneficial for the structural diversification of BBR derivatives. And all the derivatives reviewed in this article, even those synthesized by the same group, only have horizontal activity evaluation of the same series of compounds synthesized in the same period of time, but have no vertical activity evaluation of all the compounds synthesized in different periods and in different groups, which hindered the study of the SAR of BBR derivatives and the development of BBR derivatives with DNA binding effects.

In view of these, more BBR derivatives with DNA binding effects should be synthesized and the molecular diversity should be considered in the synthesis process. The binding affinities of all reported BBR derivatives should be determined at the same time, and then SAR should be summarized, so as to lay a foundation for further effective structural modifications of BBR. After several rounds of research, it is believed that the most potential BBR derivative will appear. Moreover, traditional medical theory is a valuable resource for mankind. BBR is not only an effective component in Traditional Chinese Medicine, but also a component in many Traditional Chinese Medicine prescriptions. Combining the structural modification methods of BBR with the theory of Traditional Chinese Medicine is a good and creative way to find lead compounds for DNA bindings and then for tumor treatments.

The above conclusions and perspectives can provide a valuable information for researchers devoted in developing DNA binding agents with BBR as the lead compound. It also provides a research model for the structural modifications of other natural products.
